# Body height affects the strength of immune response in young men, but not young women

**DOI:** 10.1038/srep06223

**Published:** 2014-08-28

**Authors:** Indrikis A. Krams, Ilona Skrinda, Sanita Kecko, Fhionna R. Moore, Tatjana Krama, Ants Kaasik, Laila Meija, Vilnis Lietuvietis, Markus J. Rantala

**Affiliations:** 1 Institute of Systematic Biology, University of Daugavpils, Daugavpils LV-5401, Latvia; 2 Institute of Ecology and Earth Sciences, University of Tartu, Tartu 51014, Estonia; 3 School of Psychology, University of Dundee, Dundee DD1 4HN, UK; 4 Rīga Stradiņš University, Rīga LV-1007, Latvia; 5 Pauls Stradiņš Clinical University Hospital, Rīga LV-1002, Latvia; 6 Rīga Eastern Clinical University Hospital, Rīga LV-1010, Latvia; 7 Turku Brain and Mind Center & Department of Biology, University of Turku, Turku 20014, Finland

## Abstract

Body height and other body attributes of humans may be associated with a diverse range of social outcomes such as attractiveness to potential mates. Despite evidence that each parameter plays a role in mate choice, we have little understanding of the relative role of each, and relationships between indices of physical appearance and general health. In this study we tested relationships between immune function and body height of young men and women. In men, we report a non-linear relationship between antibody response to a hepatitis-B vaccine and body height, with a positive relationship up to a height of 185 cm, but an inverse relationship in taller men. We did not find any significant relationship between body height and immune function in women. Our results demonstrate the potential of vaccination research to reveal costly traits that govern evolution of mate choice in humans and the importance of trade-offs among these traits.

It is widely assumed that larger body stature in men and greater adiposity in women reflect contrasting strategies of energy allocation in competing tissues influenced by sexual selection^1^,^2^. Males of many species have conspicuous secondary sexual traits and female mate choice is often based on these traits. It has been suggested that secondary sexual traits reliably signal male condition by acting as handicaps^3^. Development and maintenance of a large body and exaggerated secondary sexual ornaments are often energetically costly and many organisms cannot afford to allocate energy to each competing demand equally^4^. For example, if resources must be diverted away from immune function to maximize the expression of a secondary sexual trait, males may suffer increased susceptibility to parasites and pathogens^5^,^6^. Thus, only high quality males can afford to allocate resources to better immunity and attractive secondary sexual traits simultaneously (the “immunocompetence handicap hypothesis” ICHH^6^).

Humans lack extravagant secondary sexual ornaments, but mate preferences rely on substantial sexual dimorphism in body size and shape and a number of other morphological traits. Besides facial attractiveness and masculinity, physical stature is among the most important traits in human mate choice^7^,^8^. It has been proposed that these traits signal a male's testosterone and immunological status and that women's preferences for these traits may be adaptations for identifying healthy mates with good genes^9^. For example, women prefer taller men and, as a result, taller men also have higher reproductive success^10^. Tall men are perceived as having achieved higher status; perhaps reflecting dominance. However, because of human assortative mating strategies (where individuals select mates with whom they share physical, behavioural, social, and psychological similarities) women do not focus on absolute partner height^11^. In women, body size does not seem to be related either to subjective views of attractiveness^12^. Most of the studies linking facial attractiveness with indices of health did not reveal any association between general health and facial attractiveness in women^13^,^14^. However, some studies have shown that facially attractive women are healthier (e.g.^15^), which calls for more studies in this field.

To date, there has been relatively little work on a potential influence of sexual selection on body height in men and women. The aim of the research was to identify associations between body height and immune function of young men and women in the least developed area of Latvia, where trade-offs between growth and general health may affect the size of secondary sexual traits. We expected that body height of men would signal immune function as proposed by the ICHH. We did not expect such a relationship in young women since our previous study found that women's adiposity and facial attractiveness do not correlate with immune responsiveness in terms of hepatitis B antibody production^14^. The hepatitis B virus is a major global health problem. It can cause chronic liver disease and puts infected individuals at high risk of death from cirrhosis of the liver and liver cancer. More than 350 million people have chronic liver infections. More than 600 000 people die every year due to the acute or chronic consequences of hepatitis B^16^.

## Results

**Figure 1:**
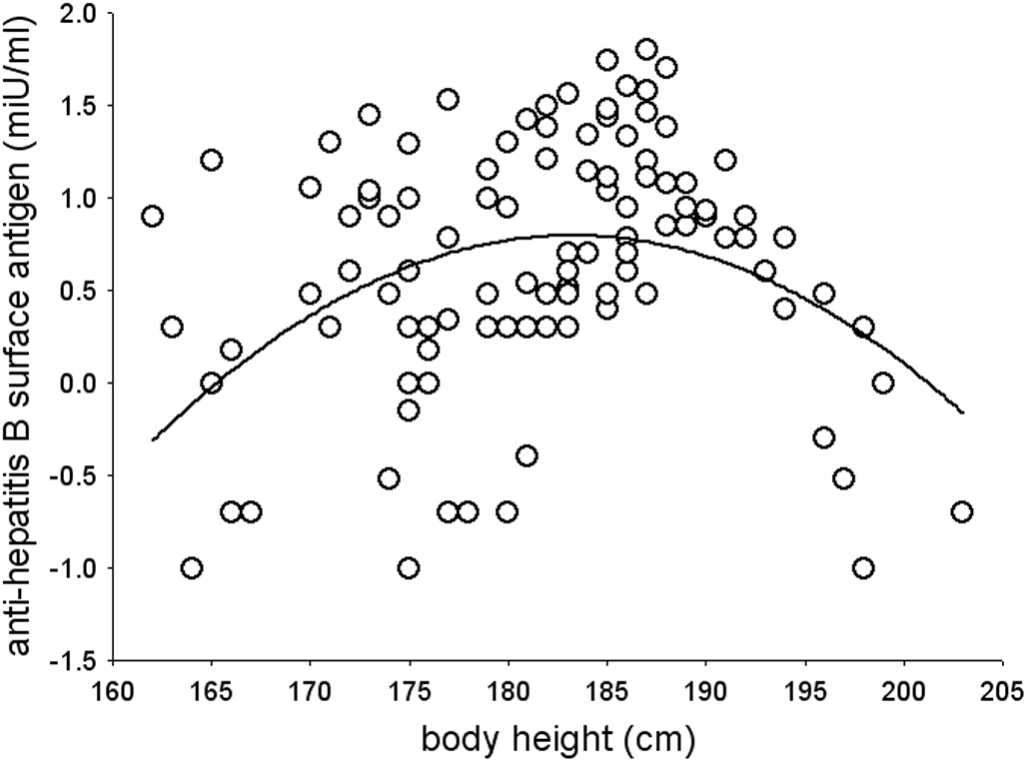
Non-linear relationships between body height and antibody response (log-transformed) in men.

We first explored bivariate relationships between body height and antibody level using a simple linear regression model. A significant positive linear relationship was found between antibody titers (mean antibody concentration, 8.31; s.d., 9.76, range 0–62 miU/ml) and height (mean height, 180.50; s.d., 9.76, range 157–203 cm) in men (β = 0.25, p = 0.004). On further inspection, the relationship between antibody response and height was non-linear (*F*_2,127_ = 19.43, *p* < 0.001). That is, there was positive relationship between height and antibody response up to 185 cm, which turned into a negative relationship in men taller than 185 cm (Fig. 1).

**Figure 2:**
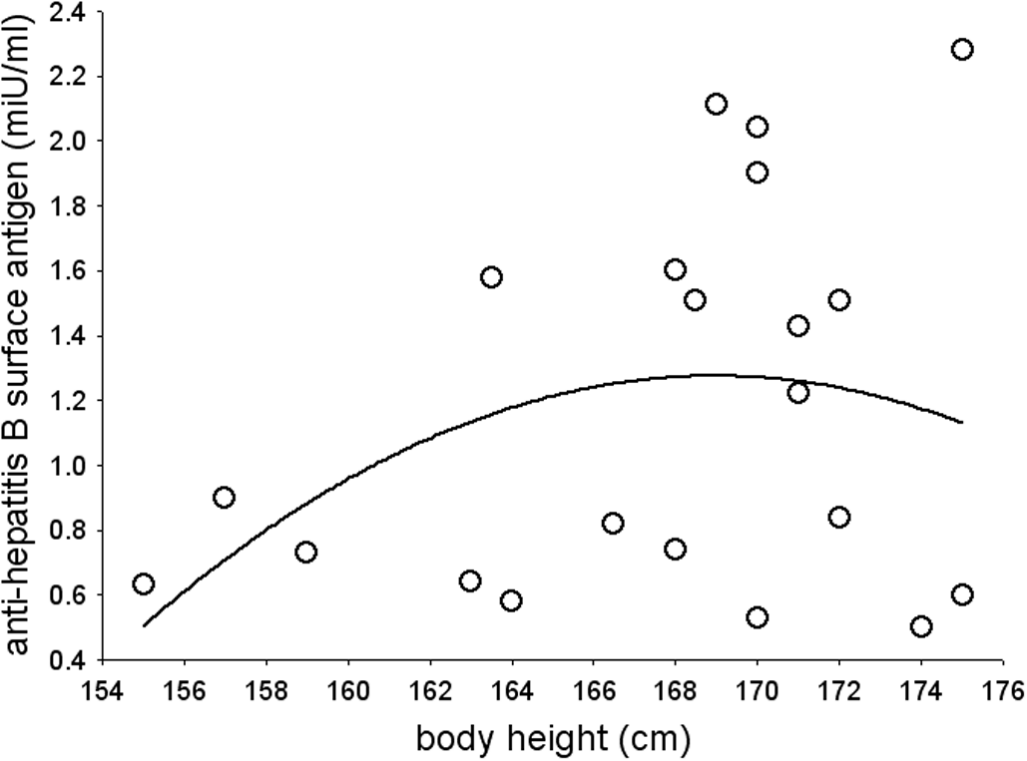
Non-linear relationships between body height and antibody response (log-transformed) in women.

We did not find any significant relationship between body height (mean height, 167.81; s.d., 6.14, range 154–183 cm) and antibody response (mean antibody concentration, 12.50; s.d., 33.47, range 0–188.60 miU/ml) in young women (*p* = 0.58). We also did not find any indication about non-linearity between antibody response and height in women (*F*_2,58_ = 1.28, *p* = 0.29, Fig. 2).

Men were significantly taller than women in our sample (one-way ANOVA, df = 194, F = 92.79, *p* < 0.001). We did not find a significant difference in the strength of immune response between men and women (one-way ANOVA, df = 189, *F* = 1.61, *p* = 0.21). However, a generalized linear model with sex as a between subjects factor and body height as a covariate revealed a significant interaction between sex and body height in predicting immune response (*F*_1,193_ = 6.68, *p* = 0.02).

## Discussion

Taller men are generally preferred by females and they also have higher reproductive success^10^. It has been suggested that height may signal status and dominance. In this study we revealed a potential additional benefit associated with stature, as height was positively related to the ability to mount an immune response against a novel antigen. Interestingly, however, this relationship was curvilinear, such that heights of above approximately 185 cm were inversely associated with antibody response. This shows that such important morphological traits as stature come with a cost. Life-history theory assumes that an individual cannot invest equal resources into all its needs, and allocation of limited resources to different functions of an organism causes numerous trade-offs between competing needs^4^. Our results suggest that building larger stature in ontogeny or supporting a large body size in adulthood might be costly in men in terms of reduced ability to mount immunity against novel infections in adulthood.

We did not find any association between body height and immune response (i.e. ability to produce antibodies) in women. A significant interaction between sex and body height in men and women suggests a sex difference in association between immune defence and body height in humans. However, this difference is not related to the mean values of immune response against the antigen of hepatitis B, which were found to be similar across sexes. While building a larger body would be beneficial not only in men but also in women, because larger bodies generally improve physical strength, larger stature might be traded-off against other important need components of women's bodies, such as maintenance of hormonal systems. However, we cannot state that building of a larger body is free of any costs in terms of immune function in women. Different components of the immune system may trade-off with each other making costs of having larger stature more difficult to detect^17^. This remains to be tested in future studies.

The efficiency of traits as signals should depend on the fact that they transmit honest information about the intrinsic quality of the bearer. ‘Good genes' models of sexual selection propose that male ornaments indicate underlying quality^3^; which can be inherited by the offspring, thereby improving their viability. This requires genetic viability in fitness-related traits. However, strong selection tends to deplete genetic variability in the traits^18^. ICHH suggests a mechanism which does not need depletion of genetic variability while linking female choice, ornaments, immune function and parasite loads of the males^5^. ICHH states that males carrying genes for superior immune function might be able to allocate more resources from their immune system to costly traits such as larger stature acting as handicaps. While many studies support the ICHH^9^,^19^, other studies have failed to do so^20^.

To resolve this paradox it has been suggested that environmental context may play a key role in determining relationship between the strength of immune system and phenotypic traits^21^. It has been recently shown that preferences for cues in men's faces related to testosterone may be dependent on a measure of societal development^22^,^23^,^24^. Thus, societal-level factors associated with environmental harshness may influence both development of many important morphological traits and also their relative value. This led us to conclude that maximum values of “cost-free” stature could differ according to the level of societal development, and we would suggest that there should be greater number of tall men in countries with greater index of societal development.

In conclusion, we found that men's body height signals an important dimension of quality, and that there are cues in men's height to immune status. We did not find any significant link between body height and antibody response in women. Furthermore, our results suggest trade-offs in the allocation of resources to height with immune function in young men.

## Methods

### Participants and measurements of body height

One hundred thirty Latvian men (mean age, 21.85; s.d., 2.49, range 19–30 years) and 65 women (mean age, 20.26, s.d. 1.29, range 18–24 years) participated in the study. Participants were students from Daugavpils University. We measured body height of participants between 8.00 and 11.00 A.M. with a precision of 0.5 cm. All participants were born in families of low to average income: workers at state plants and collective farms during the last decade under the Soviet Union, and less than 100 EUR/month income per family member after regaining independence.

### Assays of immune response

Before activation of the immune system by hepatitis-B vaccine (Engerix-B, GlaxoSmithKline), we collected venous blood in 6 ml vials to measure the presence of antibodies. Plasma was stored separately from the blood cells at −80°C for later analysis. One month after the vaccination, we again collected venous blood to measure antibodies produced. To quantitatively determine serum hepatitis B surface antigen (anti-HBs) levels, we used the commercially available AxSYM® AUSAB® microparticle enzyme immunoassay (MEIA). Anti-HBs concentrations were expressed in mIU/ml. None of the participants had hepatitis B specific antibodies before the vaccination.

### Statistical analysis

We could not detect non-normality in body height (Shapiro-Wilks test). Antibody level data, however, were not normally distributed, so the natural logarithm of these was used. Since we did not find any age-related effects (all *p* > 0.05), we excluded men's age from further analyses. Bivariate relationships between all variables were inspected using linear regression models.

### Ethical statement

The study was approved by the Research Ethics Committee of Daugavpils University (05/2012). All participants provided their written consent to participate in this study. The experiment was conducted according to the Declaration of Helsinki.
